# P273: Efficiency of clinical and laboratory criterion for the diagnosis of bacterial meningitis

**DOI:** 10.1186/2047-2994-2-S1-P273

**Published:** 2013-06-20

**Authors:** N Kurdyumova, G Danilov, M Shifrin, O Ershova, I Alexandrova, E Sherman, A Potapov

**Affiliations:** 1 ICU, Burdenko Neurosurgery Institute, Moscow, Russian Federation; 2 Neurosurgery, Neurotraumatology, Burdenko Neurosurgery Institute, Moscow, Russian Federation; 3 Medical IT Laboratory, Burdenko Neurosurgery Institute, Moscow, Russian Federation; 4 Epidemiology, Burdenko Neurosurgery Institute, Moscow, Russian Federation; 5 Microbiology, Burdenko Neurosurgery Institute, Moscow, Russian Federation; 6 Mathematics and Statistics, Kazan State University, Kazan, Russian Federation

## Introduction

Bacterial meningitis (BM) is an infectious inflammation of meninges, that may complicate the management of neurosurgical patients and should be recognized early.

## Objectives

This study was to estimate the effectiveness of existing BM diagnostic criterion (DC) and possibly improve it.

## Methods

As a DC for early diagnosis of bacterial meningitis we used combinations of any of 3 CSF markers (cytosis, neutrophils, glucose) with any of 3 other sings (body temperature, leukocytosis or leukopenia and immature white blood cells). A sample of 236 intensive care unit patients in 2010 - 2011 (324 days) were analyzed. Using the R software, we performed statistical analysis of BM cases defined by the DC and true BM diagnosed by doctors.

## Results

Our program recognized BM based on the DC. A doctor diagnosed BM considering a wider range of BM signs.

Effectiveness of DC consisted of its sensitivity, specificity and promptitude (proportion of cases detected by DC earlier).

In 28 of 32 cases with confirmed criterial diagnosis the DC was the first to identify BM (sensitivity - 84.2%, specificity - 85.9%, promptitude - 87.5%).

There were statistically significant differences in the groups of patients with confirmed and unconfirmed criterial diagnosis by the blood glucose (p = 0,01), as well as by appearance of CSF glucose in the DC (p = 0,02). Based on these results, we raised the threshold of CSF glucose in the DC (from 2.2 to 2.5 mmol/L).

The modified criterion worked out in 68 cases, the diagnosis was confirmed in 34. 29 cases of BM were diagnosed by DC earlier (sensitivity - 85.3%, specificity - 84.9%, promptitude - 85.3%).

We received no statistically significant differences between the new and the previous rates of specificity and promptitude (p = 0,86). At the same time the number of early-diagnosed cases of meningitis became one greater.

## Conclusion

The existing clinical and laboratory criterion for BM diagnostics is effective. Using this criterion improves the early diagnostics and treatment. Criterion efficiency is also possible to improve.

## Competing interests

None declared

